# Supplementary material for: An omics-based framework for assessing the health risk of antimicrobial resistance genes
Source: Nat Commun. 2021 Aug 6;12:4765. doi: 10.1038/s41467-021-25096-3 (PMC8346589; doi:10.1038/s41467-021-25096-3)
Supplement: Supplementary file 1 — Supplementary Information [file 41467_2021_25096_MOESM1_ESM.pdf]

Supplementary information

An Omics-based Framework for Assessing the Health Risk of Antimicrobial Resistance Genes

An-Ni Zhang[1-2], Jeffry M Gaston[3], Chengzhen L Dai[2], Shijie Zhao[2], Mathilde Poyet[2,4,5], Mathieu Groussin[2,4,5], Xiaole Yin[1], Li-Guan Li[1], Mark C.M. van Loosdrecht[6], Edward Topp[7], Michael R Gillings[8], William P Hanage[9], James M Tiedje[10], Katya Moniz[2], Eric J Alm[2,4,5], Tong Zhang[1,11-12]*

[1]Environmental Microbiome Engineering and Biotechnology Laboratory, The University of Hong Kong, Hong Kong, SAR, China;

[2]Department of Biological Engineering, Massachusetts Institute of Technology, Cambridge, USA;

[3]Google, Cambridge, USA;

[4]Center for Microbiome Informatics and Therapeutics, Massachusetts Institute of Technology, Cambridge, USA;

[5]The Broad Institute of MIT and Harvard, Cambridge, USA;

[6]Department of Biotechnology, Delft University of Technology, Van der Maasweg 9, 2629 HZ, Delft, the Netherlands;

[7]London Research and Development Centre (LRDC), Agriculture and Agri-Food Canada, London, Ontario, N5V 4T3, Canada;

[8]Department of Biological Sciences, Macquarie University, Sydney, New South Wales, Australia;

[9]Center for Communicable Disease Dynamics, Department of Epidemiology, Harvard TH Chan School of Public Health, Boston, United States;

[10]Department of Plant, Soil and Microbial Sciences and of Microbiology and Molecular Genetics, Michigan State University, East Lansing, MI, USA.

[11]School of Public Health, The University of Hong Kong, Hong Kong SAR, China;

[12]Center for Environmental Engineering Research, The University of Hong Kong, Hong Kong SAR, China;

*Corresponding author:

Address: Environmental Microbiome Engineering and Biotechnology Laboratory, The University of Hong Kong, Hong Kong, SAR, China.

Phone: +852-2857 8551; Fax: +852-2859 8987

Email: zhangt@hku.hk

Supplementary discussion

Risk assessment of ARGs in Structured ARG Database

To distinguish this subset of ARGs that correlated with anthropogenic impacts, we searched environmental metagenome samples and identified ARGs that were ≥100-fold more abundant in anthropogenically impacted environments than in non-anthropogenically impacted environments (Figure 1b and Supplementary Figure 2) (see Supplementary Methods and Supplementary Figure 3). We referred to ARGs that were ≥100-fold enriched in anthropogenically impacted environments as "human associated". We evaluated 2,579 putative ARGs of a total of 4050 ARGs from the Structured ARG Database[1] (referred to as ARGs of the initial set) (1,471 ARGs of the initial set could not be assessed because they were not detected in our current datasets). The presence and abundance of ARGs of the initial set were investigated in 54,718 NCBI bacterial genomes (90% aa similarity), 15,738 NCBI plasmids and other mobile genetic elements (MGEs) databases (90% aa similarity), and 854 global metagenomes (80% aa similarity) by the similarity search (searching criteria in Methods). We found that 70% (1,816) of these ARGs of the initial set were not "human associated"; and thus, were assigned to Rank IV, the lowest risk category (Figure 2b). Rank IV ARGs also showed other low risk characteristics: the majority of them were not found on any MGEs (83%, 1,505 of 1,816) (Supplementary Figure 4), and they were 5-10 times more abundant in non-anthropogenically impacted environments (Supplementary Figure 5).

We next assessed the human-associated ARGs of the initial set (763) for gene mobility (i.e. presence on plasmids, integrons, or in the intestinal microbiome mobile element database[2,3]), and for host pathogenicity (presence in the whole bacterial genomes of ESKAPE pathogens: *Enterococcus faecium*, *Staphylococcus aureus*, *Klebsiella pneumoniae*, *Acinetobacter baumannii*, *Pseudomonas aeruginosa*, and *Enterobacter* species) (Figure 2b and

Supplementary Figure 4). In total, 81% (618) of human-associated ARGs of the initial set were not carried by any mobile genetic element and were therefore classified as Rank III. The majority (84%, 122) of the human-associated, mobile genes were already present in pathogens, and we classified them as the top risk level (Rank I); 16% (23 genes) were not present in any pathogen, and were classified as Rank II.

Regulations on microbiome-based therapeutics

Regulations on live biotherapeutic products (including microbiome-based therapeutics and probiotics) usually classify them as food supplements or dietary supplements, which are subject to much less stringent regulations than pharmaceutical products. In the United States, safety of potential probiotic strains is regulated by the US-FDA in the form of the Generally Recognized as Safe (GRAS), where an antibiotic resistance profile for clinically relevant antibiotics is recommended but not required from the manufacturers[4]. In the European Union, potential probiotic strains are regulated by EFSA in the form of Qualified Presumption of Safety (QPS). EFSA issued a list of microbial cultures having QPS status, based on a series of safety assessment including acquired AMR. However, the safety assessment was conducted on each taxonomic unit of microbial cultures through literature review[5]. Although such an investigation would be useful for regulating intrinsic AMR, the significant variation of mobile AMR among bacterial species and strains was not considered. EFSA aims to detect mobile AMR *via* a multi-stage pipeline[6], but the first stage of their pipeline entails running phenotypic testing against a small subset of antibiotics (13) and discarding any strains that do not exhibit higher than expected levels of resistance for their species. This may potentially cause certain mobile resistances to remain undetected: either resistances to other untested antibiotics, or mobility not accompanied by increased levels of resistance.

Supplementary Methods

Total environmental concentration of antibiotics across environments

We performed a literature survey on the concentrations of clinical and livestock antibiotics across environments. We searched for literatures that surveyed the antibiotics of classes quinolones, sulfonamides, macrolides, tetracycline, and chloramphenicol. Raw data of the concentrations of antibiotics was collected from the tables and/or supplementary data from the original literatures (Supplementary Data 1). The concentrations of all antibiotics of a sample were summed up to represent the total concentration of antibiotics (breakdown of calculations in Supplementary Data 1 details). The total concentration of antibiotics was further normalized to the unit of ng/g (for solid samples) or ng/L (for liquid samples).

Antibiotic Resistance in Bacterial Life Tree and Global Eco-Systems

The distribution of antibiotic resistance genes (ARGs) and class 1 integrases (*intI1*) in bacterial life tree and global eco-systems was obtained from the ARGs online searching platform (ARGs-OSP)[7]. ARGs-OSP detects ARGs by searching against Structured ARG Database v1.0[1] using usearch v11.0, diamond 0.9.24, and blast 2.5.0+[8–10]. The Whole Genome Dataset (WGD) on ARGs-OSP collected 54,718 all currently available bacterial genomes (≥ 50% completeness, < 10% contamination, and curated by Genome Taxonomy Database[11]) from NCBI Genome Database. The Metagenome Dataset (MGD) on ARGs-OSP collected 854 metagenomic datasets of Illumina shotgun sequencing. The metagenomes were classified into 7 habitat-types[2, 3] based on metadata, including four undisturbed natural environments (water, sediment, soil, and permafrost) and three human-related environments (WWTPs, animal feces, and human feces). We further searched ARGs in 15,738 (all available after quality screening) NCBI plasmids (curated by PlasFlow[12]) and other MGEs databases (integrons[2] and intestinal microbiome mobile element database ImmeDB[3]).

The search cutoff was set for genomes and MGEs as e-value of 1e-5, 90% aa similarity over 80% aa hit length; and for metagenomes as e-value of 1e-7, 80% aa similarity over 75% aa hit length[1,13–15]. The bias from sequencing depth and bacterial DNA ratio across samples was controlled using Equation 1 to normalize the copy of ARGs by the total number of bacterial cells[1,14] (Supplementary Figure 11). Total bacterial cell number of one metagenomic sample was inferred by counting the average copy number of bacterial essential single copy genes (ESCMGs)[14,16]. Fecal contamination in a metagenome was evaluated by the total percentage of reads covered by three taxa as fecal markers, including uncultured crAssphage[17], *Bacteroides*[18], and *Escherichia coli*[19]. Taxonomy information of a metagenome was inferred kraken[20] using default library and settings.

$$abu_{ij} = \frac{\frac{Reads_{number_{ARGi}}}{Length_{ARGi}}}{Bacterial_{cell_{number_{samplej}}}} \text{ (Equation 1)}$$

Here, abu_{ij} represented the abundance of ARG_i in a metagenomic $sample_j$; $Reads\_number_{ARGi}$ represented the total number of metagenomic reads mapped to ARGi; $Length_{ARGi}$ represented the nucleotide gene length of ARG_i (bp); $Bacterial\_cell\_number_{samplej}$ represented total bacterial cell number in a metagenomic $sample_j$.

Information and mothertables were downloaded from ARGs-OSP to investigate the characteristics of each ARG. In details, we investigated the factors of “host taxonomy”, “host range”, “host pathogenicity”, “mobility”, and “anthropogenic enrichment” of each ARG, using self-written python and R scripts of python 3 (https://www.python.org/) and R 3.3.2[4] (packages ‘ggplot2’,’dplyr’,’ggalt’,’ggthemes’, ‘fBasics’ and ‘plyr’). Phylogenetic tree was constructed by mafft v7.4 (--nuc --adjustdirection --quiet --retree 2 --maxiterate 100) and

FastTree 2.1.10[21,22] and visualized by iTOLv5[23]. We defined the “host range” as the highest taxonomy level (from “strain” to “phylum”) shared by all the hosts of an ARG. To obtain the information for the “host pathogenicity” of one ARG, we referred to previous studies[5, 6] to predict the pathogenicity of a bacterial genome by matching and manually curating its species-level taxonomy against a list of ESKAPE pathogens. All the sequences in WGD were classified into chromosomes and plasmids by the genbank annotation and PlasFlow 1.0[8] using the default threshold of 0.7 for probability filtering. All dendrograms are visualized by *Hierarchic* or *Tree* layout in Cytoscape 3.3.0[9]. Statistics tests were conducted by R 3.3.2[4] fisher.test(alternative='greater', simulate.p.value =TRUE,B = 1000, conf.int = TRUE, conf.level = 0.95) and ks.test().

ARG Risk Ranking Framework

We designed an ARG risk ranking framework that ranks all 4,050 ARGs in SARG v1.0[1] by sequentially assessing three factors, i.e., “human associated enrichment”, “mobility”, and “host pathogenicity” (Figure 2b). The framework aimed to evaluate the potential transfer and dissemination of an ARG into human pathogens, as it represented the potential risk of an ARG to human health and the priority of an ARG to be controlled.

The framework classified the mobile and human associated enriched resistance from intrinsic and natural resistance, which could be a guidance for similarity-based databases expanded from presumptive ARGs. The framework considered not only the potential mobility by HGT (“mobility”), but also the potential ecological connectivity to human pathogens and maintenance within human pathogens by the selection of antibiotics (“human associated enrichment”).

The performance of our framework was assessed by comparing Rank I ARGs against the

known clinically relevant ARGs (Figure 3). We defined "clinically relevant ARGs" as ARGs that were reported to have caused antibiotic treatment failure and/or outbreak of multidrug resistant pathogens in hospitals. We collected a list of the "clinically relevant ARGs" that were 1) prioritized by the WHO[24] because they rendered many of antibiotic agents ineffective; or 2) reported to have caused treatment failure and/or outbreak in hospitals across the world in literatures[25–34] 3) or being widespread on MGEs in clinically relevant environments such as hospital wastewater [25,35–38].

ARG detection and risk assessment in genomes and metagenomes

We developed a bioinformatic tool arg_ranker (https://github.com/caozhichongchong/arg_ranker; pip install arg_ranker) for detecting ARGs and assessing the ARG risks in metagenomes and genomes. The arg_ranker detects ARGs in genomes and metagenomes, computes the abundance of ARGs, classifies ARGs into Rank I-IV and quantifies contribution of each risk Rank. In details, arg_ranker outputs the copy number of ARGs detected for genomes and the total copies of ARGs of each risk Rank. For metagenomes, arg_ranker computes the abundance of ARGs as the copy number of ARGs divided by the bacterial cell number or 16S copy number in the same metagenomic sample. Total bacterial cell number of one metagenomic sample is inferred by counting the number of reads mapped to bacterial genomes normalized against the average bacterial genome size of that metagenomic sample[16,20]. We define the contribution of each ARG risk Rank as the average abundance of ARGs of a risk Rank divided by the average abundance of all ARGs (Equation 2).

$$Contribution\ of\ risk\ Rank_i = \frac{\frac{\sum abu(ARGs\ of\ risk\ Rank_i)}{No.ARGs\ of\ risk\ Rank_i}}{\frac{\sum abu(all\ ARGs)}{No.\,all\ ARGs}}\ (Equation\ 2)$$

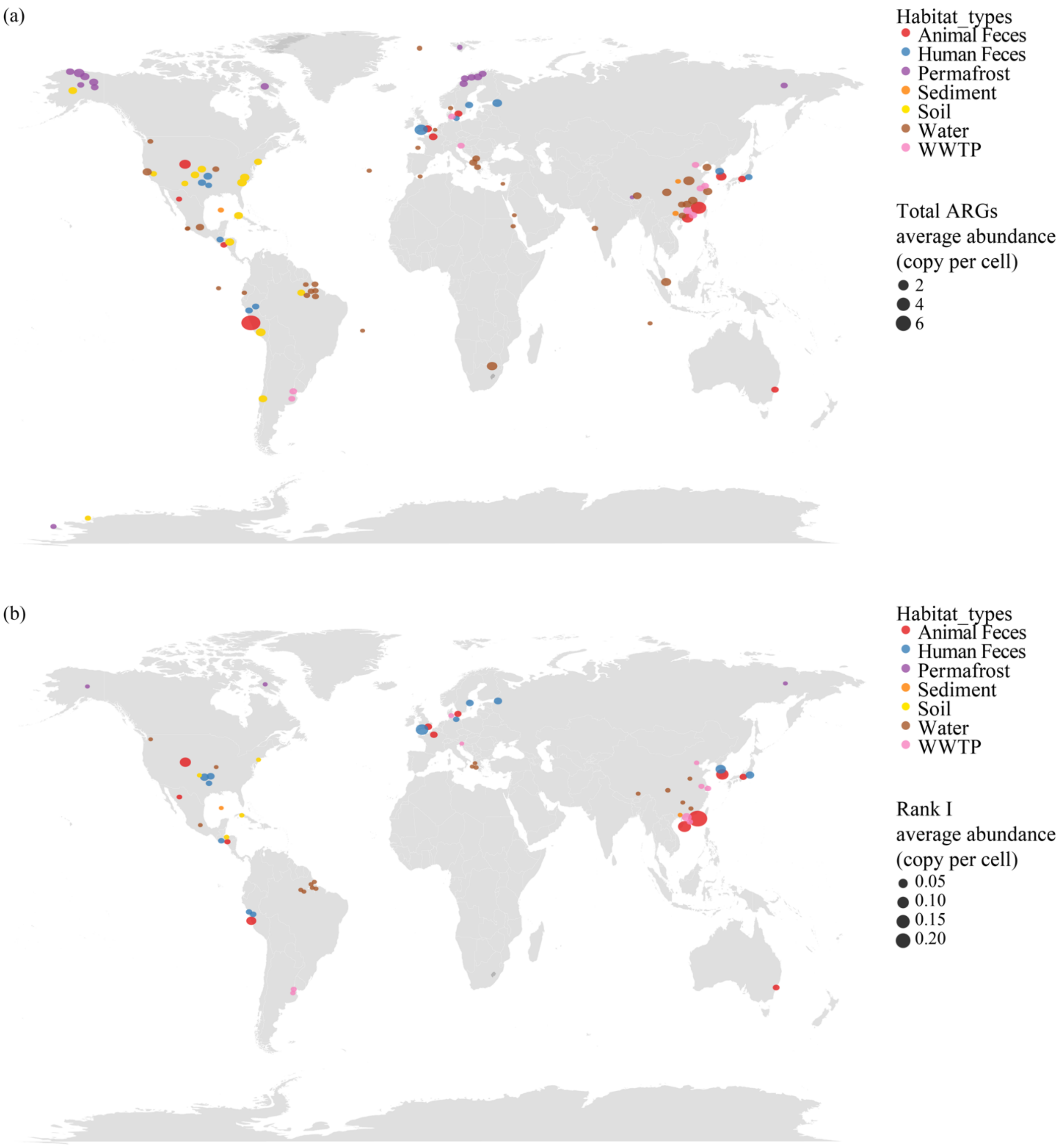

Supplementary Fig. 1. The global distribution of (a) total ARGs and (b) Rank I ARGs in metagenome datasets (MGD). Each dataset was represented as a circle node and was drawn proportional to the average abundance (copy per total number of bacterial cells, n) of ARGs. The location information was retrieved from the metadata of NCBI SRA Database. The color of each dataset represented its habitat-type. WWTP: wastewater treatment plants.

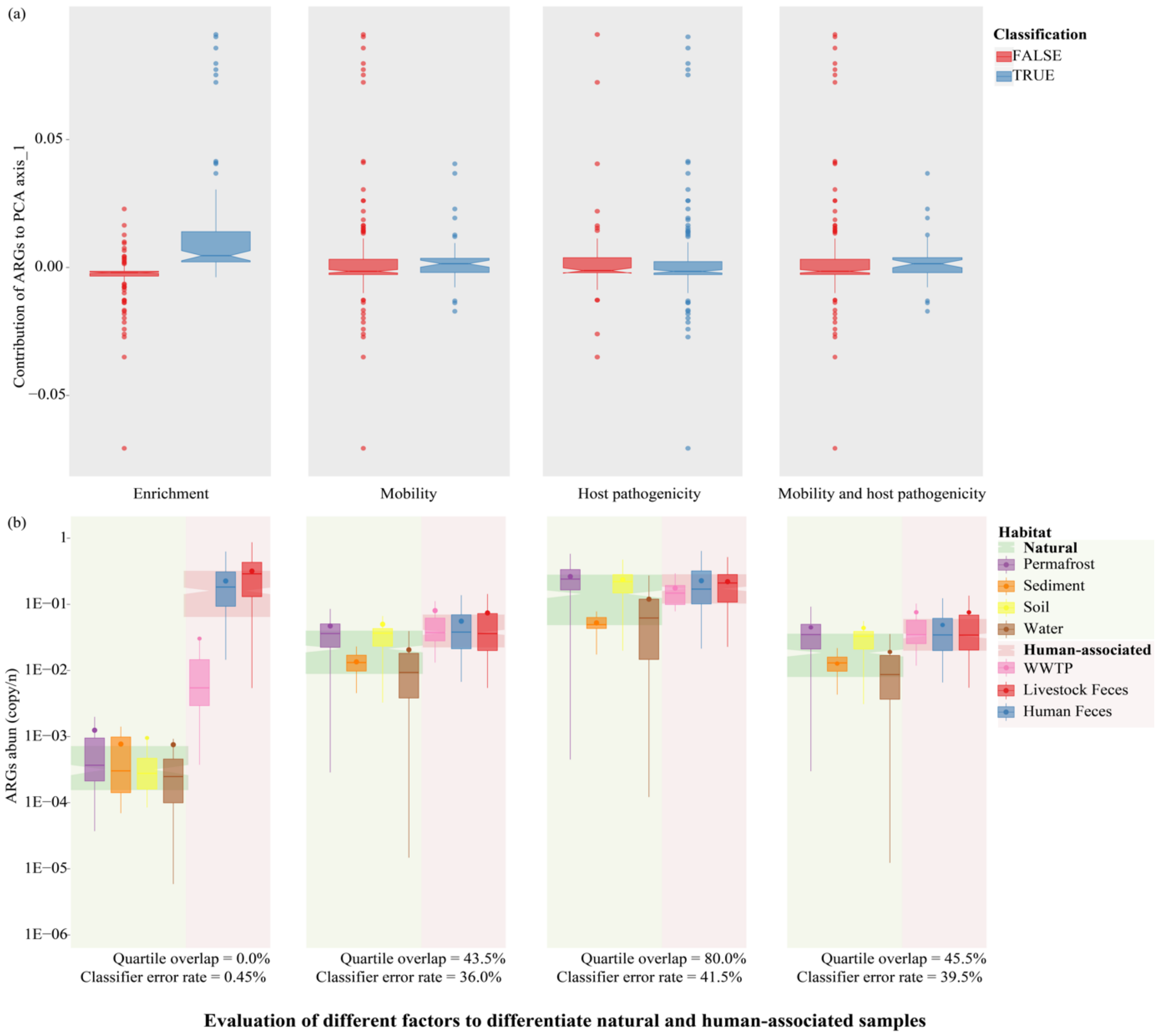

Supplementary Fig. 2. Evaluation of different factors (gene mobility, host pathogenicity, combination of gene mobility and host pathogenicity, enrichment in human-associated environments) to differentiate natural and human-associated samples by (a) contribution of different groups (True and False) of ARGs to PCA axis_1 in Figure 1c; (b) classification of samples using total abundances of ARGs qualified each factor (True group). Top 100 ARGs in each True and False group were displayed in Supplementary Fig. 3a, y_axis values were the log10 transform of the axia_1 value of their eigenvectors. Two methods were used to train the classification model:

1) Quartile overlap $= \frac{\min(Q3_{nature}-Q3_{human})-\max(Q1_{nature}-Q1_{human})}{((Q3_{nature}-Q1_{nature})+(Q3_{human}-Q1_{human}))/_2}$ (Equation 3)

2) Min classifier error rate of all linear classifier (50% is maximum, random guessing),

short for min error rate

Data are presented as mean values (dots), median values (center of box), and 25%, 75% percentiles (bounds of box). The minima and maxima represent the range of the data.

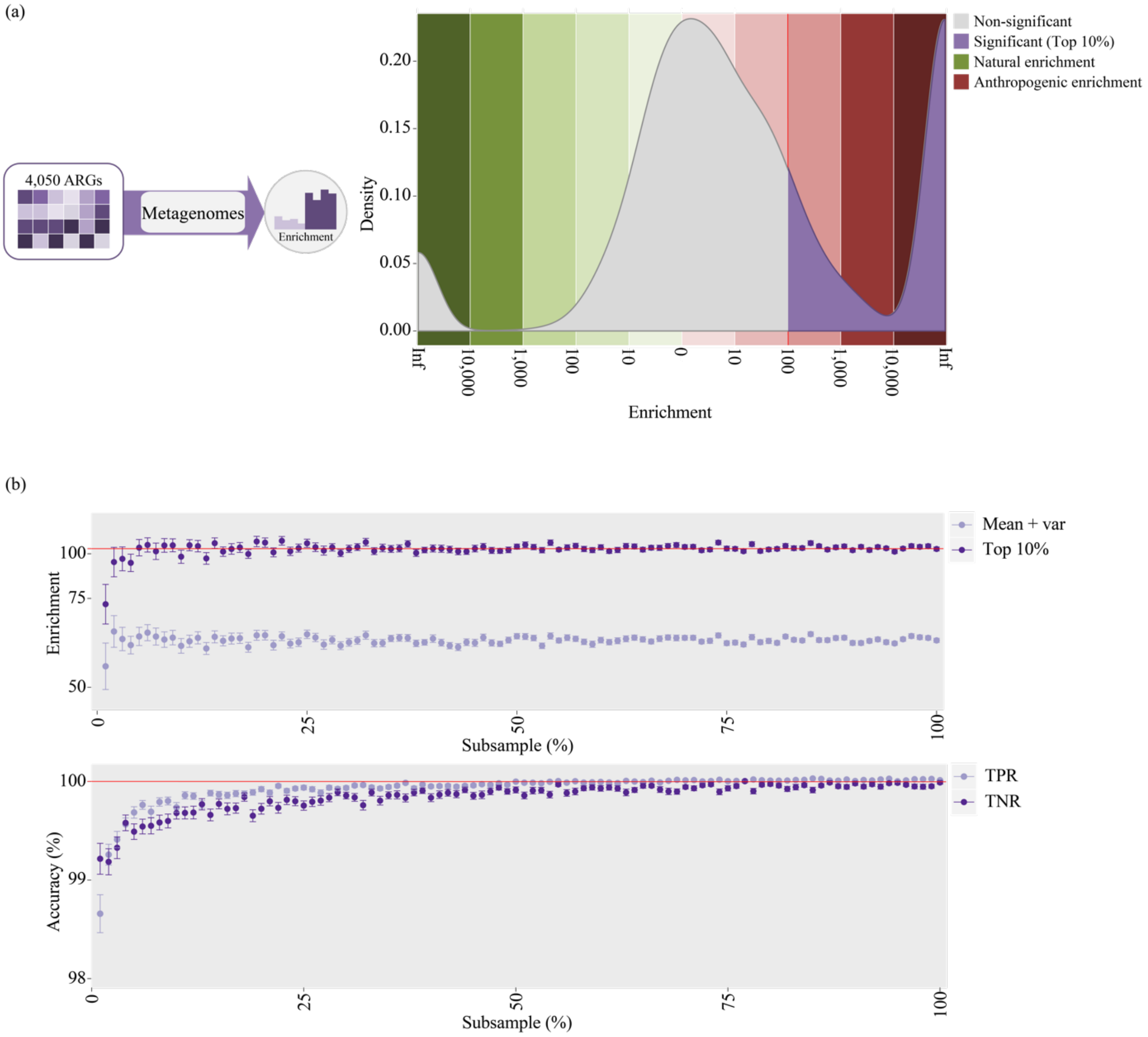

Supplementary Fig. 3. Definition of human associated enrichment (a) and evaluation of the cutoff for the significant enrichment (b). (a) Human associated enrichment was quantified as a ratio between the average abundances of the same ARG sequence in the all human associated habitats compared to all undisturbed natural habitats (Equation 4). We used 854 metagenomic samples to model the enrichment of ARG in human associated environments compared to undisturbed natural environments. The type of enrichment was represented by the color, as red

for human associated enrichment and green for natural enrichment. The “Inf” (infinity) represented that an ARG sequence was not detected in any sample from the other habitat. (b) The cutoff for significant enrichment was defined as the top 10% and was tested by sub-sampling 1% to 99% (step by 1% and iteration by 100) of the whole pool of ARGs. The cutoff (top 10%) was stable with less than 1% difference to the final cutoff when the subsampling size was larger than 30%. The cutoff (top 10%) maintained larger than the mean plus the variance of enrichment values. Of each subsample, we treated the subsampled ARGs as a training set to set the cutoff and the non-subsampled ARGs as a testing set to calculate the true positive rate (TPR, sensitivity) and true negative rate (TNR, specificity). The sensitivity and specificity were maintained as more than 99.8% when the subsampling size was larger than 30%.

$$Enrichment\ of\ ARG_i = \frac{mean(abu_{ARG_{anthropogenic}})}{mean(abu_{ARG_{nature}})}\ (Equation\ 4)$$

Data are presented as mean values +- variance (dots and error bars).

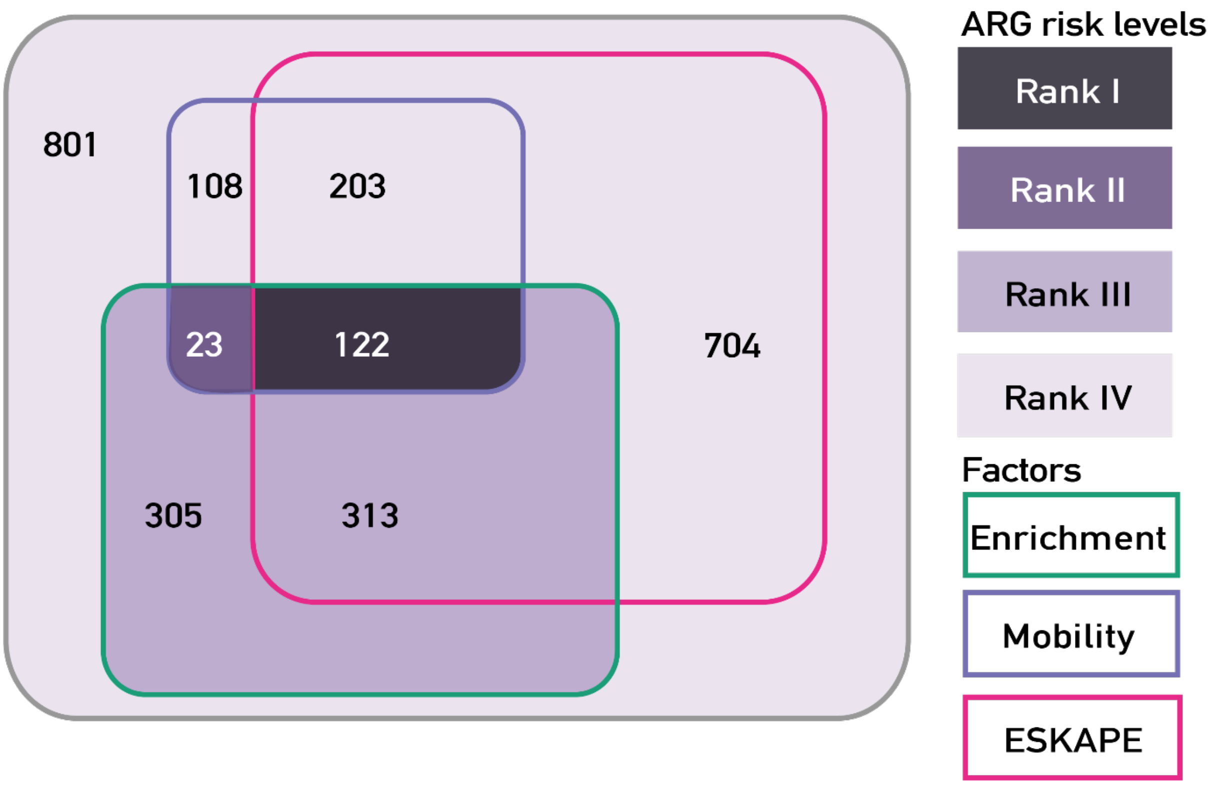

Supplementary Fig. 4. The proportion of ARG reference sequences in the Structured ARG Database v1.0 that met the factors of human-associated enrichment, gene mobility and host pathogenicity and were ranked as Rank I-IV.

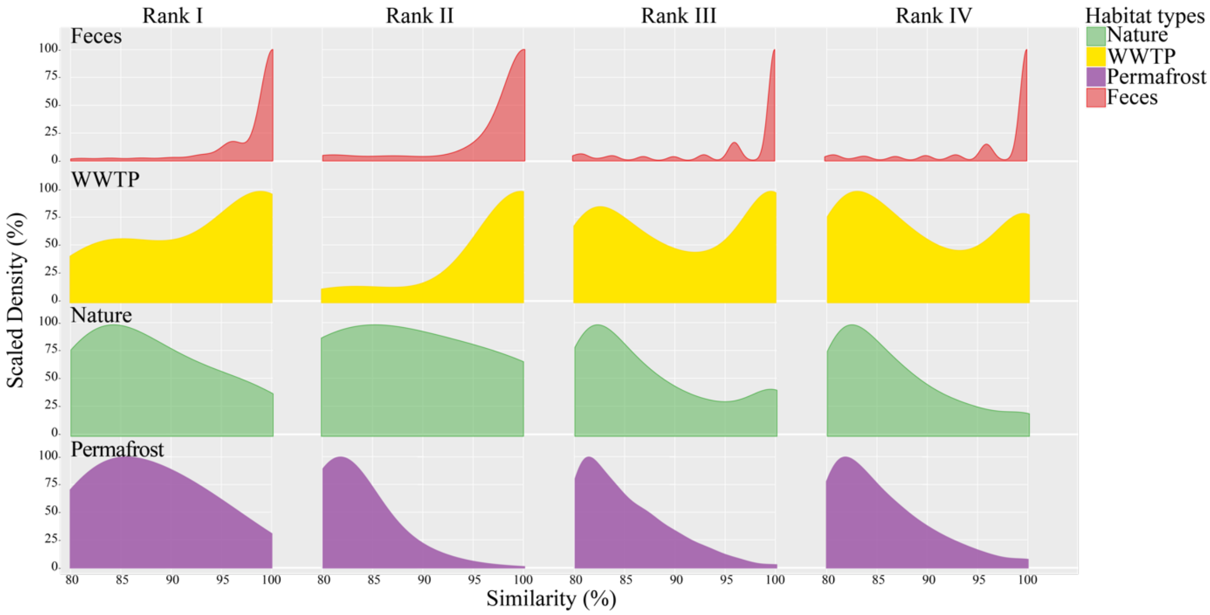

Supplementary Fig. 5. The similarities (amino acid) among sequence variants of Rank I-IV ARGs and total ARGs in different habitats. The similarities were calculated between the metagenomic sequences and the reference sequences. The density was scaled by taking the maximum density as 100%. WWTP: wastewater treatment plants.

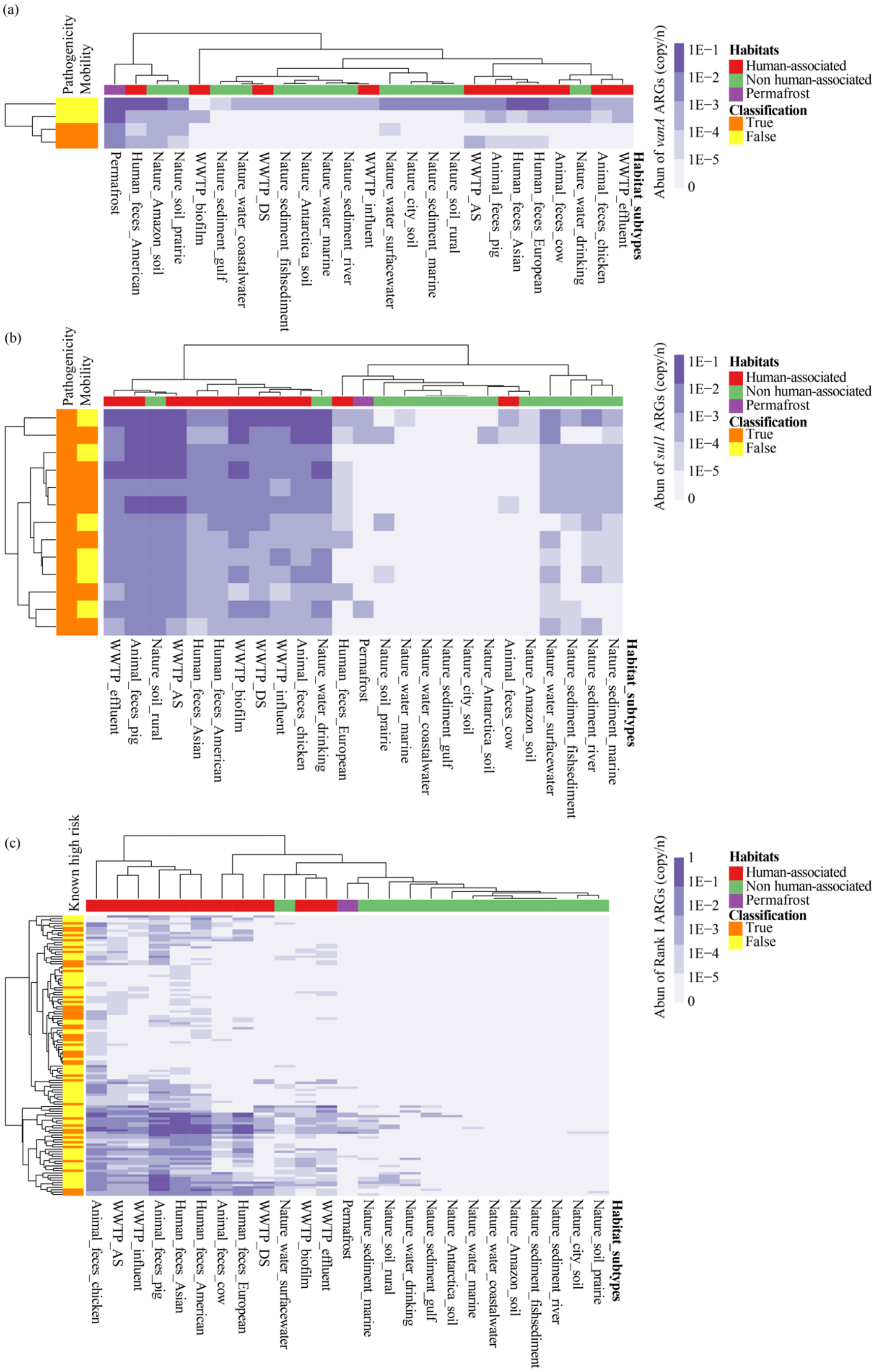

Supplementary Fig. 6. Average abundance (copy of gene per total number of bacterial cells, n) of *vanA* (a), *sul1* (b) and Rank I ARG sequences (c) in all environments. ARG sequences

(rows) and environments (columns) were clustered by Pearson correlation. For Supplementary Fig. 6a and 6b, ARG sequences were classified as True in terms of whether they were carried by pathogens (Pathogenicity), carried by mobile genetic elements (Mobility). For Supplementary Fig. 6c, ARG sequences were classified as True if there are known as high risk ARGs by previous studies.

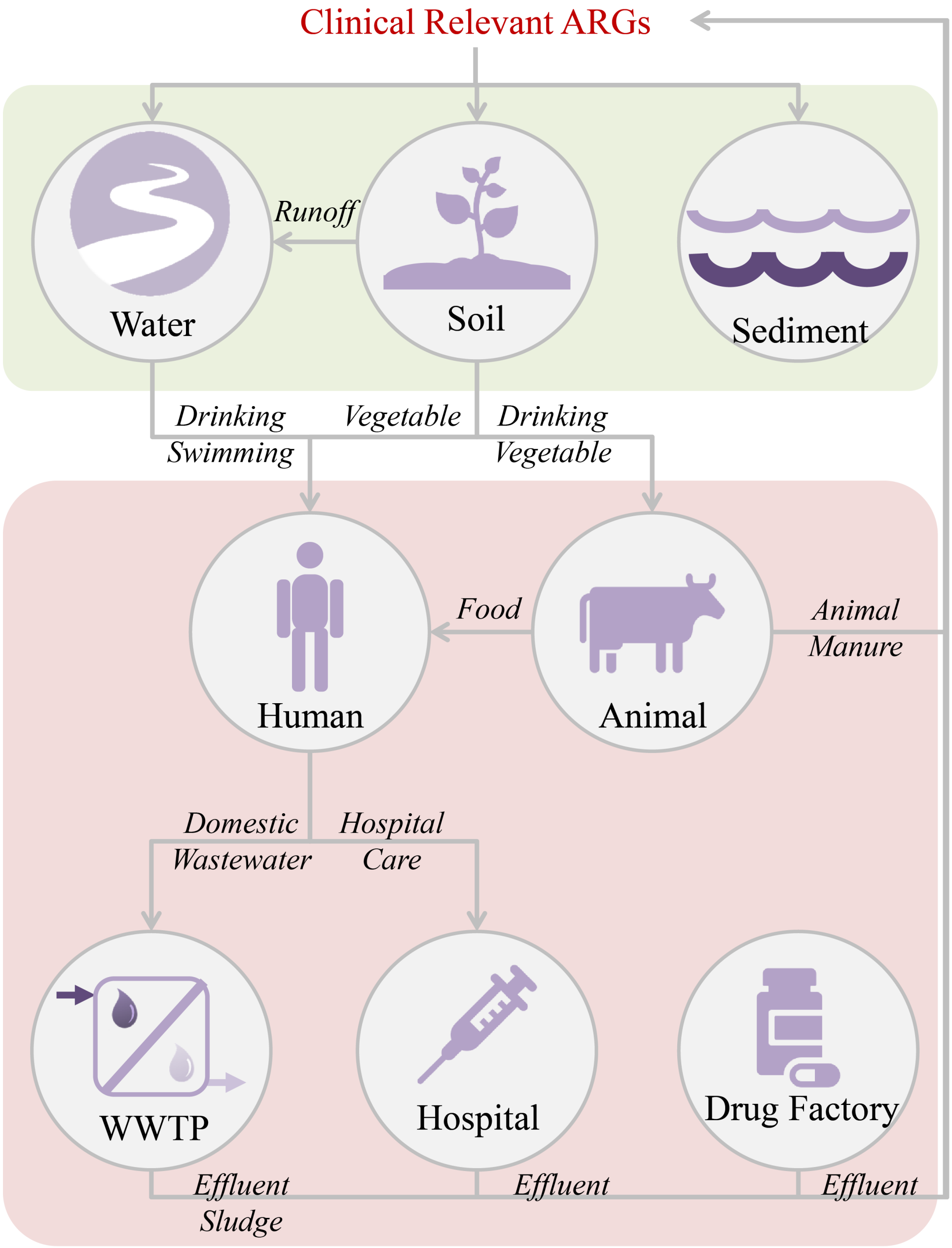

Supplementary Fig. 7. Whole cycle of antibiotic resistance dissemination in undisturbed natural and human associated environments, indicating antibiotic resistance is a growing threat to human health.

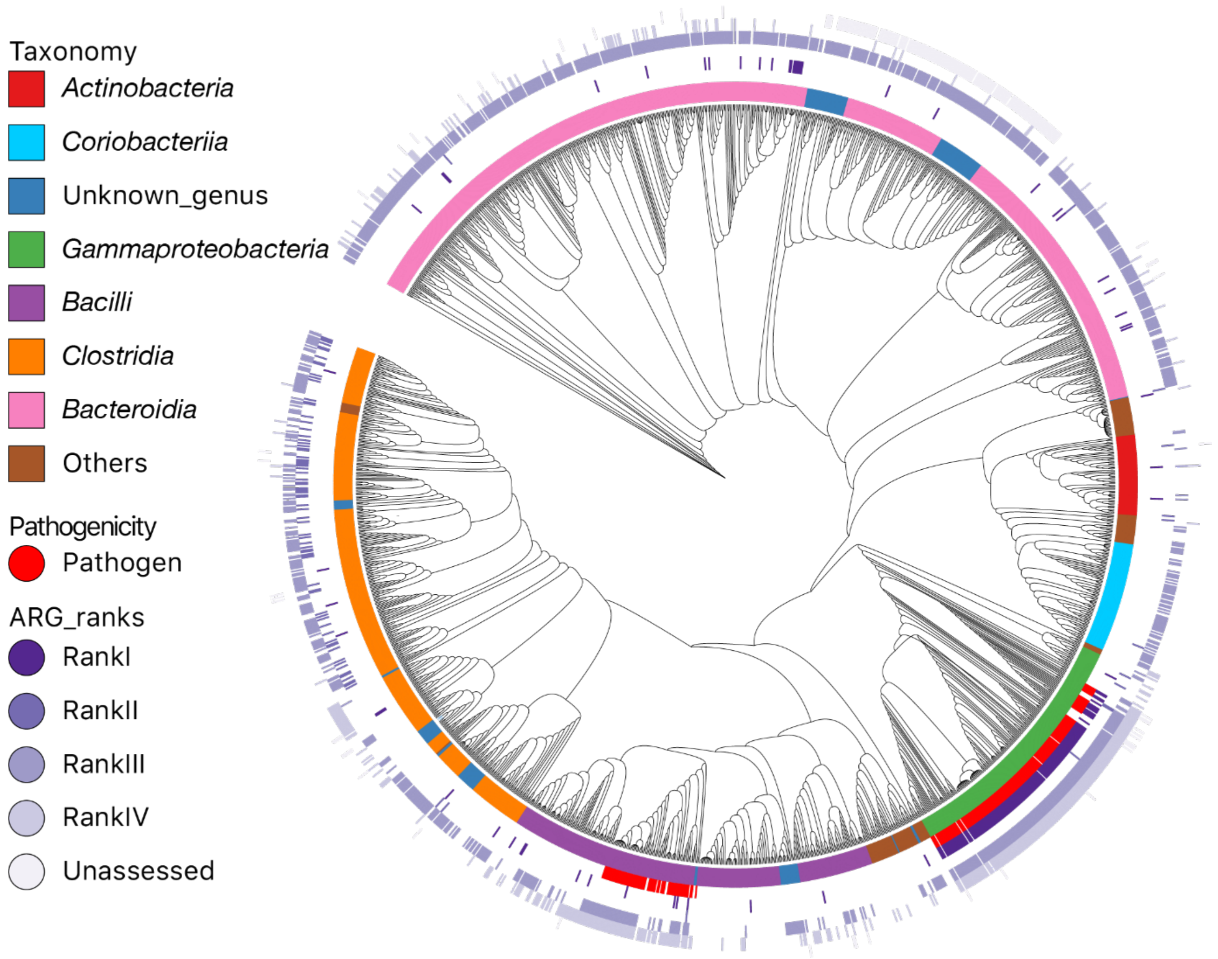

Supplementary Fig. 8. Phylogenetic tree of the 16S rRNA genes of representative human gut microbiome genomes covering 400 diverse species cultured from healthy donors who had no recent antibiotic consumption. The pathogenicity, and the presence and absence of Rank I-IV ARGs of genomes were labeled at the tips.

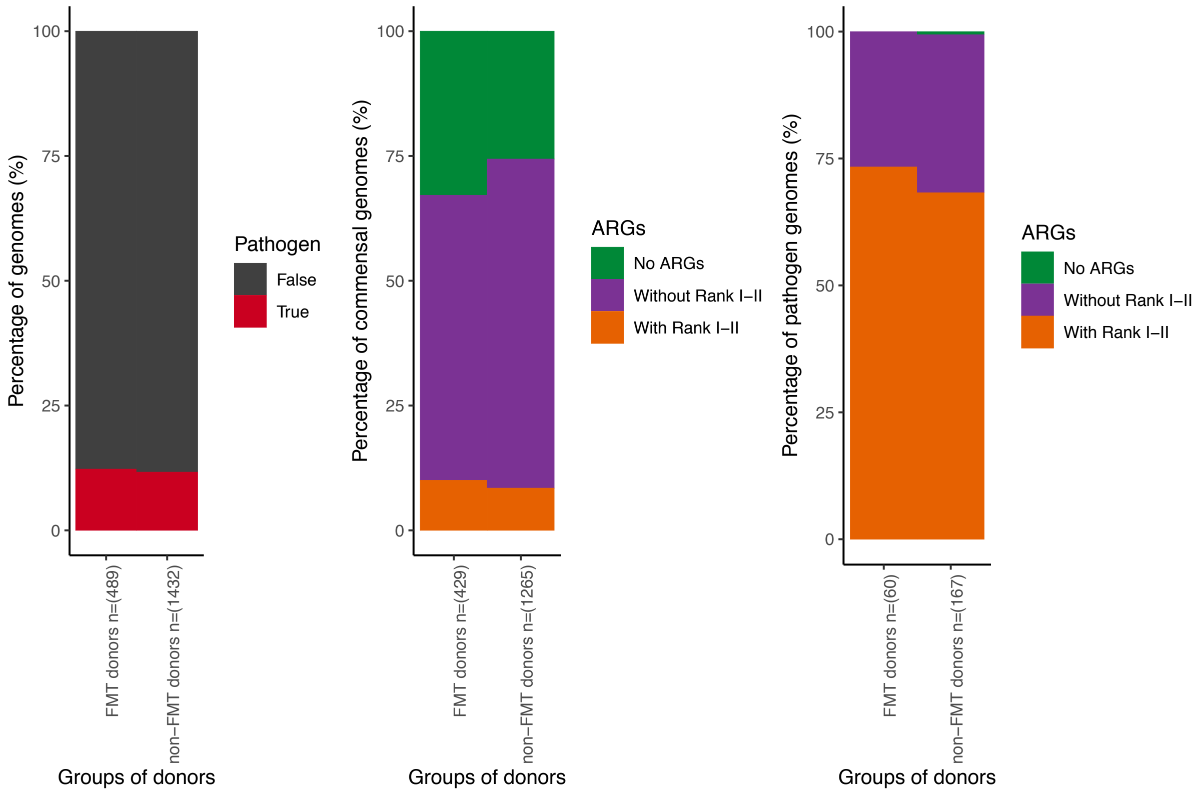

Supplementary Fig. 9. Prevalence of ARGs in representative human gut microbiome commensal (non-pathogenic) and pathogenic strains covering 400 diverse species cultured from healthy donors who had no recent antibiotic consumption. n represents the number of biologically independent donors (FMT or non-FMT human subjects).

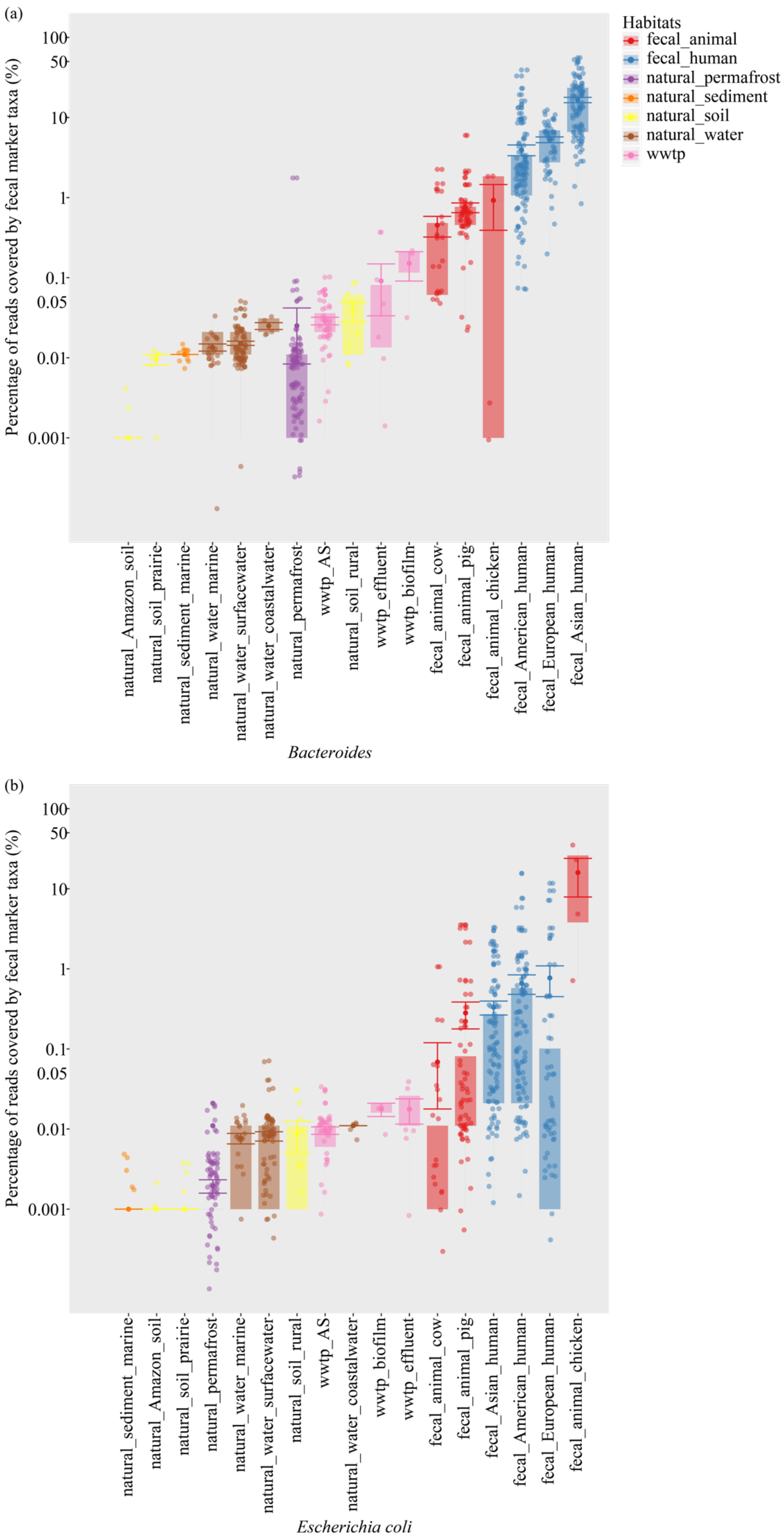
(a)
Percentage of reads covered by fecal marker taxa (%)
100
50
10
1
0.1
0.05
0.01
0.001
Habitats
fecal_animal
fecal_human
natural_permafrost
natural_sediment
natural_soil
natural_water
wwtp
natural_Amazon_soil
natural_soil_prairie
natural_sediment_marine
natural_water_marine
natural_water_surfacewater
natural_water_coastalwater
natural_permafrost
wwtp_AS
natural_soil_rural
wwtp_effluent
wwtp_biofilm
fecal_animal_cow
fecal_animal_pig
fecal_animal_chicken
fecal_American_human
fecal_European_human
fecal_Asian_human
Bacteroides
(b)
Percentage of reads covered by fecal marker taxa (%)
100
50
10
1
0.1
0.05
0.01
0.001
natural_sediment_marine
natural_Amazon_soil
natural_soil_prairie
natural_permafrost
natural_water_marine
natural_water_surfacewater
natural_soil_rural
wwtp_AS
natural_water_coastalwater
wwtp_biofilm
wwtp_effluent
fecal_animal_cow
fecal_animal_pig
fecal_Asian_human
fecal_American_human
fecal_European_human
fecal_animal_chicken
Escherichia coli

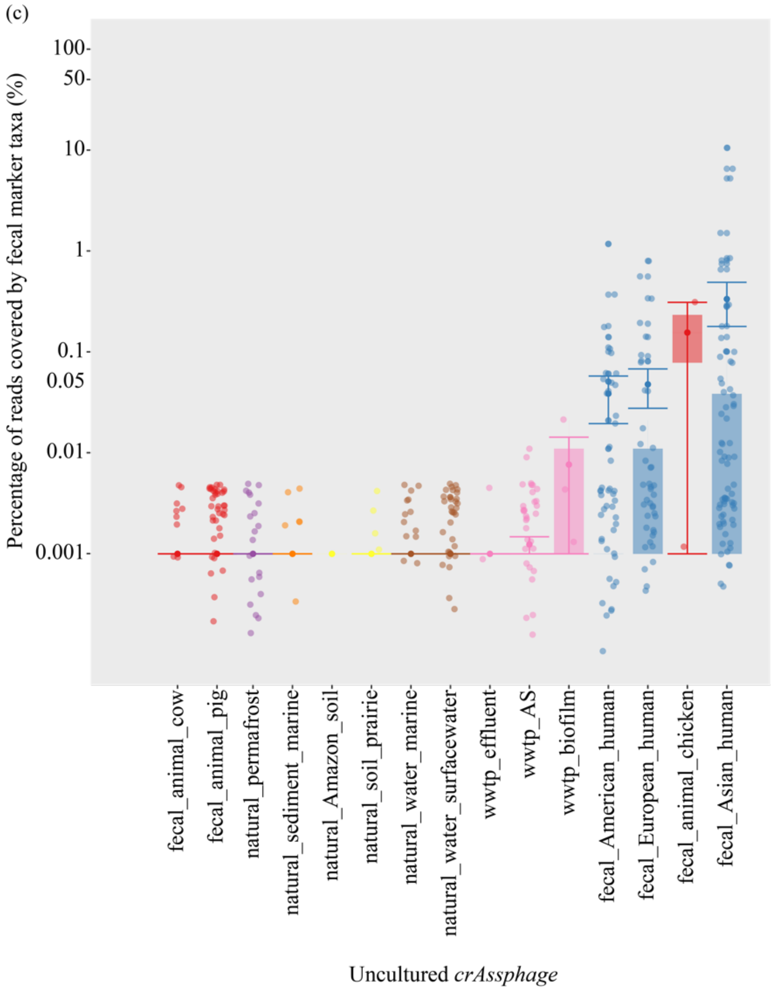

Supplementary Fig. 10. Fecal contamination of metagenomic samples represented by the total percentage of reads covered by three fecal markers. Figure a-c represent the fecal markers of *Bacteroides* (a), *Escheriachia coli* (b), and uncultured *crAssphage* (c), respectively. Non-human-associated samples were curated by less than 0.05% fecal contamination. The percentage of reads covered by three taxa was calculated by the number of reads mapping to three taxa divided by the total number of reads mapping to all taxa by kraken using default library and settings. Data are presented as mean values +- SE (dots and error bars) and 25%, 75% percentiles (bounds of box).

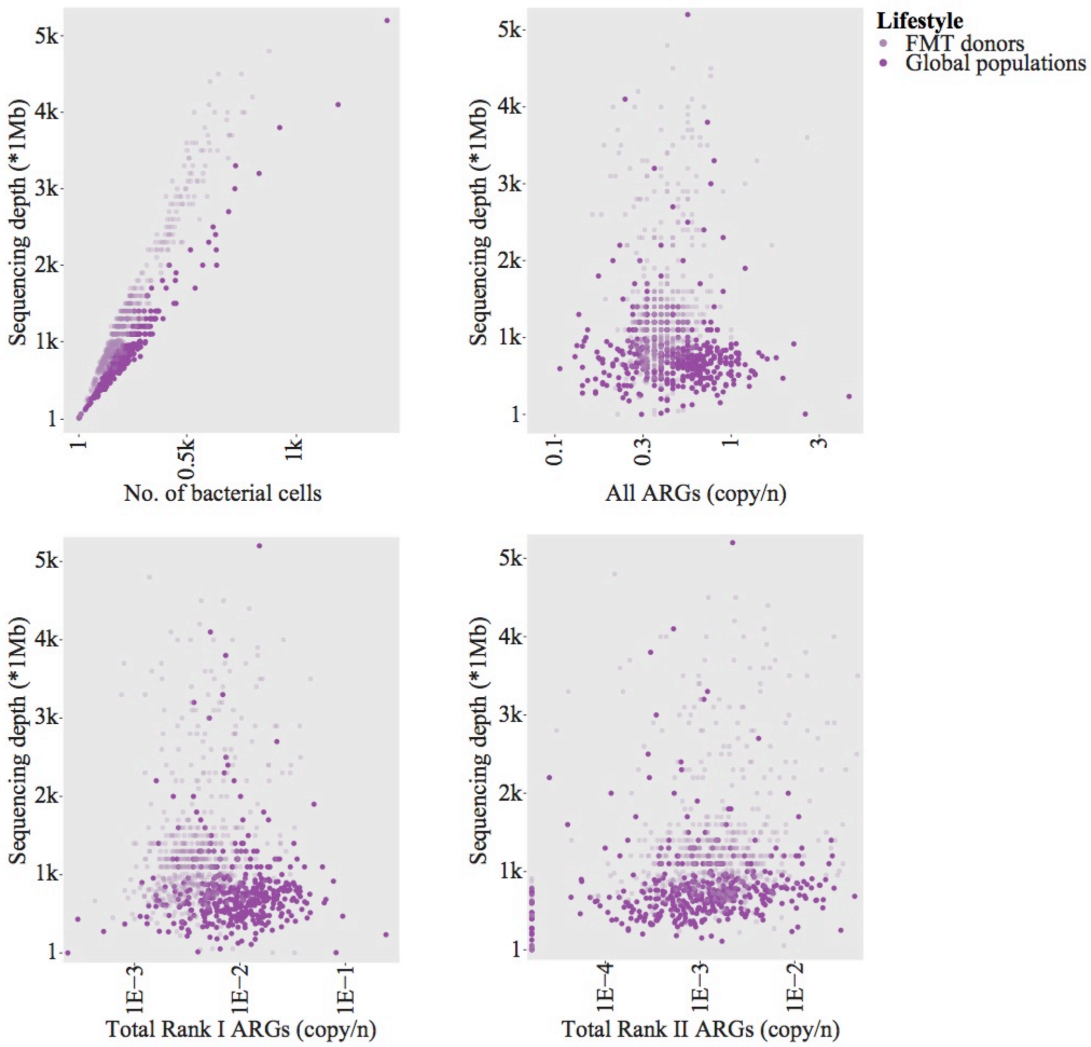

Supplementary Fig. 11. The relationship between sequencing depth and total number of bacterial cells, and abundances (copy of gene per total number of bacterial cells, n) of all ARGs, total Rank I, and Rank II. The total number of bacterial cells displayed a linear relationship to the sequencing depth and different lifestyles displayed different bacterial DNA ratio. The abundances of all ARGs, total Rank I, and Rank II displayed almost no relationship to the sequencing depth. FMT: fecal microbiota transplantation.

Supplementary References

1. Yang, Y. *et al.* ARGs-OAP: online analysis pipeline for antibiotic resistance genes detection from metagenomic data using an integrated structured ARG-database. *Bioinformatics* (2016) doi:10.1093/bioinformatics/btw136.
2. Zhang, A.-N. *et al.* Conserved phylogenetic distribution and limited antibiotic resistance of class 1 integrons revealed by assessing the bacterial genome and plasmid collection. *Microbiome* **6**, 130 (2018).
3. Jiang, X., Hall, A. B., Xavier, R. J. & Alm, E. J. Comprehensive analysis of chromosomal mobile genetic elements in the gut microbiome reveals phylum-level niche-adaptive gene pools. *PloS one* **14**, (2019).
4. *Early Clinical Trials With Live Biotherapeutic Products: Chemistry, Manufacturing, and Control Information; Guidance for Industry*. https://www.fda.gov/regulatory-information/search-fda-guidance-documents/early-clinical-trials-live-biotherapeutic-products-chemistry-manufacturing-and-control-information (2016).
5. Koutsoumanis, K. *et al.* Update of the list of QPS-recommended biological agents intentionally added to food or feed as notified to EFSA 11: suitability of taxonomic units notified to EFSA until September 2019. *Efsa Journal* **18**, (2020).
6. Additives, E. P. on *et al.* Guidance on the characterisation of microorganisms used as feed additives or as production organisms. *EFSA Journal* **16**, e05206 (2018).
7. Zhang, A. N., Hou, C.-J., Negi, M., Li, L.-G. & Zhang, T. Online searching platform for antibiotic resistome in bacterial tree of life and global habitats. *FEMS Microbiology Ecology* (2020).
8. Camacho, C. *et al.* BLAST+: architecture and applications. *BMC bioinformatics* **10**, 421 (2009).

9. Edgar, R. C. Search and clustering orders of magnitude faster than BLAST. *Bioinformatics* **26**, 2460–2461 (2010).

10. Buchfink, B., Xie, C. & Huson, D. H. Fast and sensitive protein alignment using DIAMOND. *Nat Methods* **12**, 59 (2015).

11. Parks, D. H. *et al.* A standardized bacterial taxonomy based on genome phylogeny substantially revises the tree of life. *Nature biotechnology* (2018).

12. Krawczyk, P. S., Lipinski, L. & Dziembowski, A. PlasFlow: predicting plasmid sequences in metagenomic data using genome signatures. *Nucleic Acids Res* (2018).

13. Zhang, A. N., Hou, C.-J., Li, L.-G. & Zhang, T. ARGs-OSP: online searching platform for antibiotic resistance genes distribution in metagenomic database and bacterial whole genome database. *bioRxiv* 337675 (2018).

14. Yin, X. *et al.* ARGs-OAP v2. 0 with an Expanded SARG Database and Hidden Markov Models for Enhancement Characterization and Quantification of Antibiotic Resistance Genes in Environmental Metagenomes. *Bioinformatics* **1**, 8 (2018).

15. Li, L.-G., Xia, Y. & Zhang, T. Co-occurrence of antibiotic and metal resistance genes revealed in complete genome collection. *ISME J* **11**, 651–662 (2017).

16. Nayfach, S. & Pollard, K. S. Average genome size estimation improves comparative metagenomics and sheds light on the functional ecology of the human microbiome. *Genome biology* **16**, 51 (2015).

17. Karkman, A., Pärnänen, K. & Larsson, D. J. Fecal pollution can explain antibiotic resistance gene abundances in anthropogenically impacted environments. *Nature communications* **10**, 1–8 (2019).

18. Savichtcheva, O., Okayama, N. & Okabe, S. Relationships between Bacteroides 16S rRNA genetic markers and presence of bacterial enteric pathogens and conventional fecal indicators. *Water research* **41**, 3615–3628 (2007).

19. Carlos, C. *et al.* Escherichia coli phylogenetic group determination and its application in the identification of the major animal source of fecal contamination. *BMC microbiology* **10**, 161 (2010).
20. Wood, D. E. & Salzberg, S. L. Kraken: ultrafast metagenomic sequence classification using exact alignments. *Genome biology* **15**, R46 (2014).
21. Katoh, K. & Standley, D. M. MAFFT multiple sequence alignment software version 7: improvements in performance and usability. *Molecular biology and evolution* **30**, 772–780 (2013).
22. Price, M. N., Dehal, P. S. & Arkin, A. P. FastTree 2–approximately maximum-likelihood trees for large alignments. *PloS one* **5**, e9490 (2010).
23. Letunic, I. & Bork, P. Interactive tree of life (iTOL) v3: an online tool for the display and annotation of phylogenetic and other trees. *Nucleic Acids Res* **44**, W242–W245 (2016).
24. *2019 ANTIBACTERIAL AGENTS IN CLINICAL DEVELOPMENT an analysis of the antibacterial clinical development pipeline*. https://apps.who.int/iris/bitstream/handle/10665/330420/9789240000193-eng.pdf (2019).
25. Le, T.-H. *et al.* Occurrences and characterization of antibiotic-resistant bacteria and genetic determinants of hospital wastewater in a tropical country. *Antimicrobial agents and chemotherapy* **60**, 7449–7456 (2016).
26. French, G., Shannon, K. & Simmons, N. Hospital outbreak of Klebsiella pneumoniae resistant to broad-spectrum cephalosporins and beta-lactam-beta-lactamase inhibitor combinations by hyperproduction of SHV-5 beta-lactamase. *Journal of Clinical Microbiology* **34**, 358–363 (1996).
27. Feizabadi, M. M. *et al.* Distribution of bla TEM, bla SHV, bla CTX-M genes among clinical isolates of Klebsiella pneumoniae at Labbafinejad Hospital, Tehran, Iran. *Microbial drug resistance* **16**, 49–53 (2010).

28. Coque, T. M., Oliver, A., Pérez-Díaz, J. C., Baquero, F. & Cantón, R. Genes encoding TEM-4, SHV-2, and CTX-M-10 extended-spectrum β-lactamases are carried by multiple Klebsiella pneumoniae clones in a single hospital (Madrid, 1989 to 2000). *Antimicrobial agents and chemotherapy* **46**, 500–510 (2002).

29. Yong, D. *et al.* Characterization of a new metallo-β-lactamase gene, blaNDM-1, and a novel erythromycin esterase gene carried on a unique genetic structure in Klebsiella pneumoniae sequence type 14 from India. *Antimicrob Agents Chemother* **53**, 5046–5054 (2009).

30. Betteridge, T., Partridge, S. R., Iredell, J. R. & Stokes, H. W. Genetic context and structural diversity of class 1 integrons from human commensal bacteria in a hospital intensive care unit. *Antimicrob Agents Chemother* **55**, 3939–43 (2011).

31. Lee, W. G., Jernigan, J. A., Rasheed, J. K., Anderson, G. J. & Tenover, F. C. Possible Horizontal Transfer of thevanB2 Gene among Genetically Diverse Strains of Vancomycin-Resistant Enterococcus faecium in a Korean Hospital. *Journal of clinical microbiology* **39**, 1165–1168 (2001).

32. Del Campo, R. *et al.* Detection of a single van A-containing Enterococcus faecalis clone in hospitals in different regions in Spain. *Journal of Antimicrobial Chemotherapy* **48**, 746–747 (2001).

33. Valdezate, S. *et al.* Large clonal outbreak of multidrug-resistant CC17 ST17 Enterococcus faecium containing Tn 5382 in a Spanish hospital. *Journal of antimicrobial chemotherapy* **63**, 17–20 (2008).

34. Karthikeyan, K., Thirunarayan, M. & Krishnan, P. Coexistence of bla OXA-23 with bla NDM-1 and armA in clinical isolates of Acinetobacter baumannii from India. *Journal of antimicrobial chemotherapy* **65**, 2253–2254 (2010).

35. Liu, Y.-Y. *et al.* Emergence of plasmid-mediated colistin resistance mechanism MCR-1 in animals and human beings in China: a microbiological and molecular biological study. *The Lancet infectious diseases* **16**, 161–168 (2016).
36. Martinez, J. L., Coque, T. M. & Baquero, F. What is a resistance gene? Ranking risk in resistomes. *Nat Rev Microbiol* **13**, 116–23 (2015).
37. Berendonk, T. U. *et al.* Tackling antibiotic resistance: the environmental framework. *Nat Rev Microbiol* **13**, 310–7 (2015).
38. Berglund, B. Environmental dissemination of antibiotic resistance genes and correlation to anthropogenic contamination with antibiotics. *Infection ecology & epidemiology* **5**, 28564 (2015).
