# Supplementary material for: An omics-based framework for assessing the health risk of antimicrobial resistance genes
Source: Nat Commun. 2021 Aug 6;12:4765. doi: 10.1038/s41467-021-25096-3 (PMC8346589; doi:10.1038/s41467-021-25096-3)
Supplement: Supplementary file 3 — Reporting Summary [file 41467_2021_25096_MOESM3_ESM.pdf]

nature research

Corresponding author(s): Tong Zhang

Last updated by author(s): Jul 18, 2021

# Reporting Summary

Nature Research wishes to improve the reproducibility of the work that we publish. This form provides structure for consistency and transparency in reporting. For further information on Nature Research policies, see our Editorial Policies and the Editorial Policy Checklist.

## Statistics

For all statistical analyses, confirm that the following items are present in the figure legend, table legend, main text, or Methods section.

| n/a | Confirmed | |
|---|---|---|
| ☐ | ☒ | The exact sample size (*n*) for each experimental group/condition, given as a discrete number and unit of measurement |
| ☐ | ☒ | A statement on whether measurements were taken from distinct samples or whether the same sample was measured repeatedly |
| ☐ | ☒ | The statistical test(s) used AND whether they are one- or two-sided<br>*Only common tests should be described solely by name; describe more complex techniques in the Methods section.* |
| ☐ | ☒ | A description of all covariates tested |
| ☐ | ☒ | A description of any assumptions or corrections, such as tests of normality and adjustment for multiple comparisons |
| ☐ | ☒ | A full description of the statistical parameters including central tendency (e.g. means) or other basic estimates (e.g. regression coefficient) AND variation (e.g. standard deviation) or associated estimates of uncertainty (e.g. confidence intervals) |
| ☐ | ☒ | For null hypothesis testing, the test statistic (e.g. *F*, *t*, *r*) with confidence intervals, effect sizes, degrees of freedom and *P* value noted<br>*Give P values as exact values whenever suitable.* |
| ☒ | ☐ | For Bayesian analysis, information on the choice of priors and Markov chain Monte Carlo settings |
| ☒ | ☐ | For hierarchical and complex designs, identification of the appropriate level for tests and full reporting of outcomes |
| ☒ | ☐ | Estimates of effect sizes (e.g. Cohen's *d*, Pearson's *r*), indicating how they were calculated |

*Our web collection on statistics for biologists contains articles on many of the points above.*

## Software and code

Policy information about availability of computer code

| | |
|---|---|
| Data collection | The presence and abundance of ARGs of the initial set in 54,718 NCBI bacterial genomes (downloaded on 02/19/2019), 15,738 (all available after quality screening) NCBI plasmids (downloaded on 02/19/2019), and 854 global metagenomes of Illumina shotgun sequencing (downloaded on 02/19/2019) were downloaded from ARG-OSP (https://args-osp.herokuapp.com/).<br>The validation datasets collected after 02/19/2019 were downloaded via 1) https://t.co/bdZxADGM7z (chromosomal genomes and plasmids from opportunistic pathogen isolates cultured from a tertiary hospital). 2) https://www.ebi.ac.uk/ena/browser/view/PRJEB31632 (116 metagenomes collected from the same tertiary hospital). 3) https://www.ncbi.nlm.nih.gov/pathogens/microbigge/# (all NCBI pathogen genomes submitted in the last two years).<br>The databases of mobile genetic element were available online: the integron database (Additional file 1 from https://microbiomejournal.biomedcentral.com/articles/10.1186/s40168-018-0516-2#Sec15) and immeDB database (http://immedb.gutfun.org/). |
| Data analysis | Data analysis used 1) self-written python script arg_ranker, which is published in this manuscript: https://github.com/caozhichongchong/arg_ranker; 2) usearch v11.0; 3) diamond 0.9.24; 4) blast 2.5.0+; 5) PlasFlow 1.0; 6) R 3.3.2 (packages 'ggplot2 v3.3.1','dplyr v0.8.3','RColorBrewer v1.1-2','ggtern v3.3.0', 'pheatmap v1.0.12' and 'plyr v1.8.4'); 7) python 3.7; 8) mafft v7.4; 9) FastTree 2.1.10; 10) iTOLv5; 11) Cytoscape 3.3.0. |

For manuscripts utilizing custom algorithms or software that are central to the research but not yet described in published literature, software must be made available to editors and reviewers. We strongly encourage code deposition in a community repository (e.g. GitHub). See the Nature Research guidelines for submitting code & software for further information.

## Data

Policy information about availability of data

All manuscripts must include a data availability statement. This statement should provide the following information, where applicable:

- Accession codes, unique identifiers, or web links for publicly available datasets
- A list of figures that have associated raw data
- A description of any restrictions on data availability

Details of methods, data, and scripts are all available in the Supplementary Information.
We developed a bioinformatic tool arg_ranker v2.0 (https://github.com/caozhichongchong/arg_ranker) for detecting ARGs and assessing the ARG risks in metagenomes and genomes (details in Supplementary Methods).
Source data and processed data generated in this study are publicly available online (DOI: 10.6084/m9.figshare.15001053). The ranking information of all ARGs of the initial set is available in Table S2 and sequences of Rank I-II ARGs are available in Supplementary_data1 and Supplementary_data2. The prevalence of ARGs in FMT datasets (human gut microbiome genomes and metagenomes) and clinical datasets used for validation are available in Tables S4-S10.
The presence and abundance of ARGs of the initial set in 54,718 NCBI bacterial genomes (downloaded on 02/19/2019), 15,738 (all available after quality screening) NCBI plasmids (downloaded on 02/19/2019), and 854 global metagenomes of Illumina shotgun sequencing (downloaded on 02/19/2019) were downloaded from ARG-OSP (https://args-osp.herokuapp.com/).
The validation datasets collected after 02/19/2019 were downloaded via 1) https://t.co/bdZxADGM7z (chromosomal genomes and plasmids from opportunistic pathogen isolates cultured from a tertiary hospital). 2) https://www.ebi.ac.uk/ena/browser/view/PRJEB31632 (116 metagenomes collected from the same tertiary hospital). 3) https://www.ncbi.nlm.nih.gov/pathogens/microbigge/# (all NCBI pathogen genomes submitted in the last two years).
The databases of mobile genetic element were available online: the integron database (Additional file 1 from https://microbiomejournal.biomedcentral.com/articles/10.1186/s40168-018-0516-2#Sec15) and immeDB database (http://immedb.gutfun.org/).

# Field-specific reporting

Please select the one below that is the best fit for your research. If you are not sure, read the appropriate sections before making your selection.

☐ Life sciences ☐ Behavioural & social sciences ☒ Ecological, evolutionary & environmental sciences

For a reference copy of the document with all sections, see nature.com/documents/nr-reporting-summary-flat.pdf

# Ecological, evolutionary & environmental sciences study design

All studies must disclose on these points even when the disclosure is negative.

| | |
|---|---|
| Study description | To identify 'high-risk' antibiotic resistance genes (ARGs) that may pose significant safety concerns for microbiome-based therapeutics, we designed a framework by which different ARGs may be classified into different risk categories on the basis of their potential to contribute to the emergence of new or multi-drug resistance in pathogens. The framework is an easy-to-implement decision tree that uses the factors of human-associated enrichment, gene mobility, and host pathogenicity. Data covering diverse habitats, diverse bacteria taxa and mobile genetic elements, and diverse human individuals was collected to evaluate the risk of each ARG in terms of the factors of human-associated enrichment, gene mobility, and host pathogenicity. |
| Research sample | This study used publicly available data collected before 02/19/2019, consisting of 54,718 curated high-quality bacterial genomes, 15,738 (all available after quality screening) NCBI plasmids (curated by PlasFlow) and other MGEs databases (all available integrons and intestinal microbiome mobile element database ), 854 metagenomic datasets of Illumina shotgun sequencing representing four natural environments (water, sediment, soil, and permafrost) and three human-related environments (WWTPs, animal feces, and human feces), 1,921 representative human gut microbiome genomes cultured from 59 healthy donors, and 563 human gut microbiome metagenomes of 84 healthy fecal microbiota transplantation (FMT) donors. To validate our framework, we analyzed three testing datasets collected from 02/20/2019 and 02/10/2021 whose data were not included in the training datasets collected before 02/20/2019. Dataset1 contains the chromosomal genomes and plasmids from opportunistic pathogen isolates cultured from a tertiary hospital, which samples were collected from 179 sites associated with 45 hospital beds over 1.5 years (downloaded from https://t.co/bdZxADGM7z). Dataset1 covers a total of 94 taxa (2,347 genomes and 5,910 plasmids) (Tables S6 and S7). Dataset2 contains all 116 metagenomes collected from the same study of Dataset1 (by platform Illumina HiSeq 2000, accession numbers ERX3237365-ERX3237728, ERX3667056-ERX3667128, ERX3669272-ERX3669296) downloaded from https://www.ebi.ac.uk/ena/browser/view/PRJEB31632 (Table S8). Dataset3 contains all NCBI pathogen genomes submitted in the last two years, which were downloaded from https://www.ncbi.nlm.nih.gov/pathogens/microbigge/# with "collection date" between 02/20/2019 to 02/10/2021 (Table S9). Dataset2 covers a total of 362 taxa (42,481 genomes). |
| Sampling strategy | All samples were downloaded from a published study of ARGs OSP (Zhang, A. N., Hou, C.-J., Negi, M., Li, L.-G. & Zhang, T. Online searching platform for antibiotic resistome in bacterial tree of life and global habitats. FEMS Microbiology Ecology (2020)). We included all publicly available NCBI bacterial genomes (downloaded on 02/19/2019) (after a screened by > 50% completeness, <10% contamination), 15,738 all available NCBI plasmids (downloaded on 02/19/2019). |
| Data collection | An-Ni Zhang collected all data of bacterial genomes, mobile genetic elements, environmental metagenomes, human gut microbiome genomes and metagenomes from FMT donors, and validation datasets by 1) NCBI Batch Entrez https://www.ncbi.nlm.nih.gov/sites/batchentrez; 2) antibiotic resistance genes (ARGs) Online Searching Platform version 1 (ARGs OSP v1.0): https://args-osp.herokuapp.com/; 3) https://www.ebi.ac.uk/ena/browser/view/PRJEB31632; 4) https://t.co/bdZxADGM7z; 5) https://www.ncbi.nlm.nih.gov/pathogens/microbigge/#. |

| | |
|---|---|
| Timing and spatial scale | The training datasets were collected on 02/19/2019 and the testing datasets were collected on 02/10/2021 including data sampled from 02/20/2019 and 02/10/2021, which were not included in the training datasets. |
| Data exclusions | We excluded 1 FMT donor who had consumed antibiotics in the six months prior to sample collection. |
| Reproducibility | To validate our framework, we analyzed three datasets collected from 02/20/2019 and 02/10/2021 (Figure 4a, see Methods) whose data were not included in the training datasets (collected before 02/20/2019) that were used to develop the framework. All attempts to repeat the experiment were successful. |
| Randomization | We randomly sub-sampled 1% to 99% (step by 1% and iteration by 100) of the whole pool of ARGs to test the cutoff for the factor "human-associated enrichment". Of each subsample, we treated the subsampled ARGs as a training set to set the cutoff and the non-subsampled ARGs as a testing set to calculate the true positive rate (TPR, sensitivity) and true negative rate (TNR, specificity). |
| Blinding | The data used in this study was collected by previous studies to test various hypotheses that were not relevant to the hypothesis in this study. |

Did the study involve field work? ☐ Yes ☒ No

# Reporting for specific materials, systems and methods

We require information from authors about some types of materials, experimental systems and methods used in many studies. Here, indicate whether each material, system or method listed is relevant to your study. If you are not sure if a list item applies to your research, read the appropriate section before selecting a response.

## Materials & experimental systems

| n/a | Involved in the study |
|---|---|
| ☒ | ☐ Antibodies |
| ☒ | ☐ Eukaryotic cell lines |
| ☒ | ☐ Palaeontology and archaeology |
| ☒ | ☐ Animals and other organisms |
| ☒ | ☐ Human research participants |
| ☒ | ☐ Clinical data |
| ☒ | ☐ Dual use research of concern |

## Methods

| n/a | Involved in the study |
|---|---|
| ☒ | ☐ ChIP-seq |
| ☒ | ☐ Flow cytometry |
| ☒ | ☐ MRI-based neuroimaging |
