# Supplementary material for: An omics-based framework for assessing the health risk of antimicrobial resistance genes
Source: Nat Commun. 2021 Aug 6;12:4765. doi: 10.1038/s41467-021-25096-3 (PMC8346589; doi:10.1038/s41467-021-25096-3)
Supplement: Supplementary file 4 — Description of Additional Supplementary Files [file 41467_2021_25096_MOESM4_ESM.pdf]

Description of Additional Supplementary Files

File Name: Supplementary Data 1

Description: The total concentration of antibiotics in different habitats collected from literature reviews.

File Name: Supplementary Data 2

Description: Sequences (amino-acid) of Rank I ARGs in SARG Database v1.0.

File Name: Supplementary Data 3

Description: Sequences (amino-acid) of Rank II ARGs in SARG Database v1.0.

File Name: Supplementary Data 4

Description: The risk ranks of all ARGs in SARG Database v1.0.

File Name: Supplementary Data 5

Description: The list of ARGs that were not found previously in pathogens (i.e. before 02/20/2019) and were newly present in pathogens in a more recent dataset (i.e. after 02/20/2019).

File Name: Supplementary Data 6

Description: The summary of presence and absence of ARGs of the initial set in 1,921 representative human gut microbiome genomes covering 400 diverse species cultured from 59 healthy donors who had no recent antibiotic consumption.

File Name: Supplementary Data 7

Description: Presence and absence of ARG sequences of the initial set in 1,921 representative human gut microbiome genomes covering 400 diverse species cultured from 59 healthy donors who had no recent antibiotic consumption.

File Name: Supplementary Data 8

Description: Presence ARGs of the initial set in human gut microbiome metagenomes of FMT donors.

File Name: Supplementary Data 9

Description: Presence ARGs of the initial set in hospital chromosomes genomes (Dataset1).

File Name: Supplementary Data 10

Description: Presence ARGs of the initial set on hospital plasmids (Dataset1).

File Name: Supplementary Data 11

Description: Presence ARGs of the initial set in hospital metagenomes (Dataset2).

File Name: Supplementary Data 12

Description: Presence ARGs of the initial set in new NCBI pathogens (Dataset3).
